# The diversity of sandflies (Psychodidae: Phlebotominae) and the presence of *Leishmania* spp. DNA in potential vectors of the Mbaracayú Forest Biosphere Reserve, Canindeyú, Paraguay: New records and findings

**DOI:** 10.1371/journal.pntd.0013806

**Published:** 2025-12-10

**Authors:** Miriam Soledad Rolón, Antonieta Rojas de Arias, Myriam Velázquez, Milena Britos, Paola Arze Selich, Jorge Alfonso Ruiz Díaz, Oscar Salvioni Recalde, Stefania Fraenkel Cálcena, Ana Gómez Garay, Nidia Martínez Acosta, María Celeste Vega Gómez

**Affiliations:** 1 Centro para el Desarrollo de la Investigación Científica (CEDIC), Asunción, Paraguay; 2 Fundación Moisés Bertoni (FMB), Asunción, Paraguay; 3 Departamento de Entomología, Servicio Nacional de Erradicación del Paludismo SENEPA - Ministerio de Salud Pública y Bienestar Social, Av. Pettirossi esq. Brasil, Asunción, Paraguay; Egerton University, KENYA

## Abstract

Leishmaniasis is a zoonotic disease where parasites of the genus *Leishmania* are transmitted among hosts by sandfly vectors of the subfamily Phlebotominae. In Paraguay, studies on sandfly biodiversity are limited, even though the disease is present in its tegumentary and visceral forms. This pioneering ecoepidemiological study analyzes the diversity, abundance, and detection of *Leishmania* spp. DNA in sandflies from primary, secondary, and degraded forest habitats within the Mbaracayú Forest Biosphere Reserve. Over a year of sampling, 954 sandflies were captured, comprising 599 females and 355 males, belonging to 12 species, 10 of which are reported for the first time in Canindeyú. The species with the highest positivity for *Leishmania* spp. DNA were the *Evandromyia cortelezzii* complex (20.3%), *Pintomyia monticola* (18.8%), *Brumptomyia brumpti* (17.4%), *Brumptomyia avellari* (15.9%), and *Psathyromyia lanei* (11.6%). In contrast, *Evandromyia evandroi*, *Micropygomyia quinquefer*, *Nyssomyia neivai*, and *Migonemyia migonei* displayed rates of 4.3% or lower, while *Psathyromyia shannoni* complex, *Brumptomyia guimaraesi*, and *Evandromyia termitophila* were negative. The identified *Leishmania* species were *L. amazonensis* (65.2%) and *L. infantum* (34.8%). Additionally, dogs and their ectoparasites from an indigenous community were analyzed, detecting *L. amazonensis* DNA in 18 flea groups (54.5%) and one tick (16.7%). However, all dogs tested negative for the Rk39 test. This study highlights the risk of leishmaniasis transmission in forested and degraded ecosystems, which could have significant public health consequences, calling for deeper analyses to assess associated risks.

## Author’s summary

The interaction between natural ecosystems and human activities in the Mbaracayú Forest Biosphere Reserve reveals a complex leishmaniasis transmission network that challenges traditional control strategies. This study uncovers a remarkable diversity of sandflies, coupled with the detection of *Leishmania* spp. DNA in vectors and dog ectoparasites, highlighting unexplored ecoepidemiological dynamics in the region. The findings emphasize the potential role of previously underestimated species in the transmission of *L. amazonensis* and *L. infantum*, even in the absence of traditionally incriminated vectors. The detection of *L. amazonensis* DNA in dog ectoparasites underscores a complex and multifaceted transmission process, where domestic and wild reservoirs interact within a delicate ecological balance. Furthermore, ecotourism activities in the region, particularly during peak vector activity seasons, could inadvertently increase human exposure to pathogens. Therefore, a one health approach is vital to integrate surveillance, environmental management, and community education measures to mitigate disease expansion and protect both people and the ecosystems they inhabit.

## 1 Introduction

Leishmaniasis is a public health issue in 98 countries distributed across four continents (the Americas, Europe, Africa, and Asia), with mandatory reporting in 32 countries, including Paraguay [1]. It is estimated that 350 million people are at risk of contracting the infection, with an annual occurrence of 700,000–1 million new cases of its various clinical forms over the last 20 years [1]. In the Americas, leishmaniasis manifests as two main clinical forms: American visceral leishmaniasis (AVL) and American tegumentary leishmaniasis (ATL), which can present in cutaneous, mucocutaneous, and diffuse forms [2,3].

Leishmaniasis is a zoonotic, vector-borne disease with a complex transmission cycle involving a diverse array of parasites, reservoirs, and vectors. The disease is caused by protozoan parasites of over 20 *Leishmania* species, transmitted primarily through the bites of infected female phlebotomine sandflies (Psychodidae: Phlebotominae), hematophagous insects with nocturnal or crepuscular habits [1]. Sandflies exhibit great diversity and a wide geographic distribution, with over 1,000 species described worldwide. Of these, 555 (538 extant and 17 fossil) species are found in the Americas [4] and *Leishmania* spp. has been detected in 60 species [5].

Following Theodor’s 1948 classification of phlebotomine sand flies, in which American species were divided into two genera, *Brumptomyia* and *Lutzomyia*, several authors have included the species considered vectors of *Leishmania* in the latter genus; however, *Lutzomyia* species were divided into several genera, and many of them into subgenera, in the classification proposed in 1995 by Galati [4]. Adopting Galati’s classification, Paraguay’s most recent official data report 29 species from 10 different genera, three of them (*Pintomyia*, *Evandromyia* and *Psathyromyia*) with different subgenera [6,7]. This anthropophilic sandfly species is widely distributed across the eastern region of Paraguay and in the western region’s Boquerón department [6]. Species reported locally as responsible for ATL transmission include *Nyssomyia neivai*, *Nyssomyia whitmani*, *Migonemyia migonei*, and *Pintomyia pessoai*, all of which are widely distributed, as well as *Psathyromyia shannoni*, reported only in the eastern region [6].

In Paraguay, several *Leishmania* species have been identified from patient samples [8]. *Leishmania infantum* has been isolated in cases of visceral leishmaniasis while various species [9–11], including *Leishmania braziliensis* [12,11], *Leishmania lainsoni* [13,14], *Leishmania amazonensis* [15], and *Leishmania guyanensis* have been identified in tegumentary forms [15,16]. Additionally, two cases of *Leishmania* major-like parasites were reported in 1994 [17].

In the zoonotic transmission cycle, dogs are key reservoir hosts for *L. infantum*, highlighting their public health importance [18,19]. However, *L. infantum* is not the only species involved in zoonotic transmission [20], as other *Leishmania* species,including *L. braziliensis, L. amazonensis, L. guyanensis, Leishmania mexicana, Leishmania panamensis, Leishmania peruviana, Leishmania naiffi, Leishmania major, Leishmania tropica and Leishmania tarentolae*, have been detected in dogs in various regions worldwide, particularly in the Americas [21–25].

Although sandflies are the only recognized biological vectors of *Leishmania* parasites, alternative transmission modes have been discussed and speculated. In areas where cases of leishmaniasis have been reported without the presence of primary vectors [26,27] or even without any described Phlebotominae species, alternative infection routes have been suggested [28]. Various non-vector transmission pathways have been proposed for humans [29–32] and dogs, including sexual transmission [33,34] and transmission through the bites of infected fleas or ticks or their accidental ingestion [35,36].

Scientific studies on the biodiversity of phlebotomine sandflies in Paraguay remain limited, with the first nationwide mapping reported in 2021 [6]. Despite this advancement, significant gaps persist regarding the natural infection status and the identification of *Leishmania* species present in sandflies classified as confirmed, suspected, or potential vectors. To date, only one study in Paraguay has employed PCR for sandfly analysis, detecting *L. infantum* DNA for the first time in *Evandromyia cortelezzii* [37].

To contribute to the understanding of the transmission cycle of leishmaniasis in the northeast of the country, we have conducted this study in the Mbaracayú Forest Natural Reserve *(RNBM by its Spanish abbreviation)*, a central area of the Mbaracayú Forest Biosphere Reserve *(RBBM by its Spanish abbreviation),* located in the Department of Canindeyú, which borders Brazil to the north and east. This department, along with San Pedro, reports the highest number of notified cases of ATL in the country, with Canindeyú also being the only department with a high number of cases across all age categories [38]. The RNBM presents ideal conditions for conducting this type of study, as it is not only a protected wilderness area but also hosts research, education, and ecotourism activities. Surrounding communities of indigenous peoples enter the reserve for hunting [39]. These characteristics foster the interaction between humans and wildlife, including vectors and reservoirs, thereby increasing the risk of transmission of various diseases.

Detailed information on the presence and distribution of parasites in vectors and reservoirs is critical to understanding the transmission cycles and dynamics of leishmaniasis in endemic areas. This knowledge is essential for developing effective control strategies.

Therefore, our primary objective was to study eco-epidemiological aspects, such as the diversity, abundance, and presence of *Leishmania* spp. DNA in sandflies across different habitats in the RNBM. Additionally, we focused on studying the presence of *Leishmania* spp. DNA in dogs and their associated ectoparasites within an indigenous community settled in the RBBM.

## 2 Materials and methods

### 2.1 Ethics statement

The collection of fleas, ticks, and blood from dogs was carried out with the authorization of the Ministry of the Environment and Sustainable Development (MADES - Paraguay) under scientific collection permits N° 036/2020. Canine samples were collected with informed consent from dog owners. Sampling was performed in accordance with the ethical standards of the Declaration of Helsinki.

### 2.2 Study area

**Fig 1:**
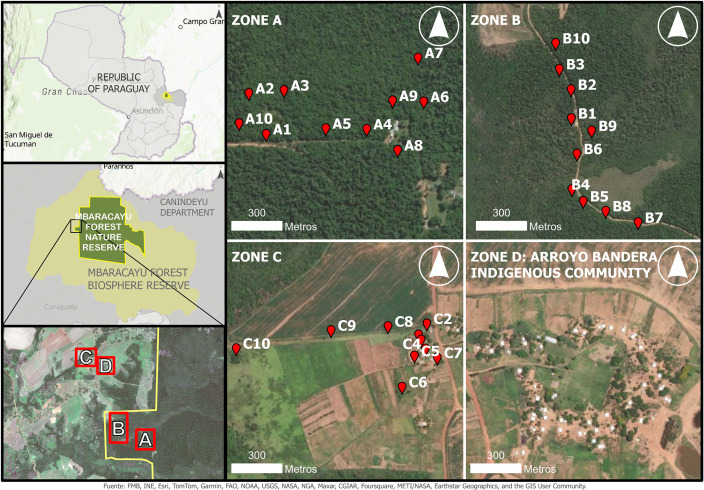
Study area in South America and location of sampling points for sandflies (zone A, B, C), dogs and ectoparasites (zone D) in different ecotopes of the RBBM, Canindeyú, Paraguay. This map was created using ArcGIS software by Esri and data from the Cartographic Atlas of Paraguay (GIS data set). ArcGIS and ArcMap are the intellectual property of Esri and are used herein under license. Copyright Esri. All rights reserved. World Imagery: https://services.arcgisonline.com/ArcGIS/rest/services/World_Imagery/MapServer. Basemaps: World Topographic Map https://cdn.arcgis.com/sharing/rest/content/items/7dc6cea0b1764a1f9af2e679f642f0f5/resources/styles/root.json.

The study was conducted in the Mbaracayú Forest Biosphere Reserve (RBBM), located in the Department of Canindeyú, northeastern Paraguay (Fig 1). This reserve includes a core area known as the Mbaracayú Forest Nature Reserve (RNBM), which covers 64,405 hectares of protected private wilderness area, managed by Fundación Moisés Bertoni (FMB). Only research, ecotourism, and subsistence hunting by the Aché indigenous people using traditional methods are permitted within this reserve [39]. The climate in the region is typically continental, classified as temperate rainy according to Köppen. The average temperature ranges between 21 and 22°C, with frosts occurring between June and October. There are two distinct seasons: a dry and cold season, and a wet and hot season, with large daily temperature fluctuations. The average annual precipitation is 1,800 mm, with the highest rainfall recorded between October and March [39].

### 2.3 Collection sites

The entomological sampling design was carried out by stratifying the collection areas into three distinct environments based on the conditions of their natural surroundings (primary forest, secondary forest, and modified areas with significant human intervention). Ten sites were selected within each of the defined strata, totaling 30 sampling units in the study area. All geographic coordinates of the sampled sites were recorded using a global positioning system (GPS) (Garmin eTrex10 and Trimble Juno D).

For sandfly capture, 20 sentinel sites were selected in the RNBM, distributed across two different ecotopes. Ten CDC-type traps were placed in a secondary forest area (Zone A: traps A1-A10), and ten traps were placed in a primary forest area (Zone B: traps B1-B10) (Fig 1). Additionally, 10 sentinel sites were selected in the RBBM, where 10 traps were placed in a degraded area (Zone C: traps C1-C10), where anthropogenic activities occur (Fig 1).

Vegetation classification was based on studies by Naidoo and Hill [40] where, the primary forest consists of high forest, bamboo forest, and liana forest, while the secondary forest is composed of riparian vegetation, bamboo understory, high forest, and bamboo forest. The degraded zone is a 20-hectare area altered by human activity, primarily dedicated to small-scale agricultural production, and is composed of meadow, pasture, degraded land, and grassland vegetation.

For the study of dogs and their ectoparasites, the Arroyo Bandera indigenous community was selected. This community is located in the RBBM, 2 km away from the RNBM (Fig 1), and forms part of their ancestral territory, where they continue to engage in hunting and gathering activities. The Aché community consists of 31 households, 169 inhabitants, and 50 dogs that roam freely and do not receive veterinary care [41].

### 2.4 Sandfly collection and taxonomical identification

Sandfly capture was carried out using CDC mini light traps (BioQuip, CA 90220, USA) [42]. Ten traps were placed in a rotating manner in Zones A, B, and C over two consecutive nights, from 6 pm to 6 am, once a month from October 2020 to October 2021 (except April 2021). The traps were positioned at distances of approximately 100–150 meters from each other, 1.5 meters above the ground. The captured sandflies were transported under cold conditions to the RNBM laboratory, where they were frozen at -20°C for two hours for subsequent identification.

Taxonomic identification of the sandflies was performed using the taxonomic keys of Galati [43]. with the generic abbreviations proposed by Marcondes [44]. Sandflies were separated by sex using a stereomicroscope (SMZ-171, Motic), and the female specimens were mounted on glass slides for microscopic observation of the genital tract and head structures (palps and cibarium). Each dissected specimen was stored individually in tubes with 70% ethanol, transported under cold conditions, and frozen at -20°C until DNA extraction.

### 2.5 Collection and taxonomic identification of fleas and ticks from domestic dogs

Fleas and ticks were collected from 40 domestic dogs belonging to the inhabitants of the Arroyo Bandera indigenous community. The dogs were sampled in May 2021, after obtaining informed consent from the community. All specimens were collected using entomological forceps and placed individually in vials without preservatives. They were then classified morphologically under a magnifying lens using taxonomic keys for fleas [45–47] and ticks [48]. Once classified, they were stored in tubes with 95% ethanol and frozen at -20°C until DNA extraction.

### 2.6 DNA extraction from sandflies, ticks, and fleas for *Leishmania* detection

The processing of female sandflies was performed individually using the thorax and abdomen of each specimen [49]. Ticks were processed individually [50], while fleas were processed individually or grouped into pools of 2–4 specimens [36], depending on the infestation level of each animal.DNA extraction was carried out using the GeneJET Genomic DNA Purification Kit (K0722, Thermo Scientific), following the manufacturer’s instructions. The purity of the extracted genetic material was evaluated using a spectrophotometer (DeNovix DS-11FX+).

### 2.7 Molecular detection of *Leishmania* DNA

To detect *Leishmania* spp., DNA extracted from sandflies, ticks, and fleas was analyzed. The primers LSGITS1-F193 (CATTTTCCGATGATTACAC) and LSGITS1-R1 (CGTTATGTGAGCCGTTATC) were used, designed to amplify a fragment of the internal transcribed spacer 1 (ITS-1) of ribosomal RNA. These primers generate fragments ranging from 220–275 bp, depending on the species of *Leishmania* [51]. For amplification, the Mix GoTaq Green Master Mix (Promega) was used in a final volume of 50 µL, containing 4 µL of DNA (25 ng/µL), 200 µM of each dNTP, 1.5 mM of MgCl_2_, 2 units of Taq polymerase, and 25 pmol of each primer. The samples were amplified in a thermal cycler (MultiGene OptiMax, Labnet) with the following conditions: initial denaturation at 95°C for 2 minutes, followed by 34 cycles of denaturation at 95°C for 20 seconds, annealing at 53°C for 30 seconds, and extension at 72°C for 1 minute. This was followed by a final extension at 72°C for 6 minutes [52]. Positive controls for the PCR reaction included DNA extracted from *L. infantum* (MHOM/BR/1972/LD), *L. braziliensis* (MHOM/CO/88/UA301), and *L. amazonensis* (IFLA/BR/67PH8). Negative controls were included in all reactions. Amplicons were visualized on a 1.5% agarose gel stained with SYBR Safe DNA Gel Stain (Invitrogen), with a 100 bp DNA ladder (Gene Ruler, Thermo Scientific) as a molecular weight size standard.

### 2.8 Identification of *Leishmania* species by sequencing

For the molecular determination of *Leishmania* species, PCR products were purified using the commercial GeneJET PCR Purification Kit (Thermo Scientific), following the manufacturer’s instructions. A volume of 20 µL of the purified product was subjected to sequencing using the Sanger method, performed by Macrogen (Geumcheon-gu, Seoul, Korea). The sequences were analyzed using MEGA7 software by comparisons with sequences deposited in the GenBank database using the Basic Local Alignment Search Tool (BLAST, http://www.ncbi.nlm.nih.gov/BLAST), considering the most similar organism according to an identity of at least 95% for species level.

### 2.9 rK39 rapid diagnostic test for canine visceral leishmaniasis

A total of 40 domestic dogs of different breeds, sex, and ages belonging to the inhabitants of the Arroyo Bandera indigenous community were analyzed. After obtaining authorization from the owners, data pertaining to the dogs were recorded in a database, and serum samples were collected. Veterinarians extracted 2 mL of blood from the central vein of each dog, and characteristics and clinical signs compatible with leishmaniasis were recorded. For the rK39 RDT (Kalazar Detect Canine Rapid Test; Inbios), 20 µL of serum were placed on the test strips and incubated with the running buffer, following the manufacturer’s instructions. The results were read after 10 minutes and were interpreted by the same person. The rK39 RDT strips were provided by the National Zoonosis Control Program and the National Rabies Center (Ministry of Public Health and Social Welfare). The serological survey results were provided to the program for monitoring and control of the canine population in the community.

### 2.10 Environmental variables around sentinel sites

To characterize the environmental conditions surrounding the sentinel sites, we used Sentinel-2A MSI satellite imagery with a spatial resolution of 10 m. Image selection followed two criteria: (i) cloud cover ≤ 10% and (ii) correspondence with the study period. For each trap, buffer zones with radii of 25 m, 50 m, and 100 m were established. Within these buffers, two vegetation and water-related indices were calculated:

*NDVI (Normalized Difference Vegetation Index):*


$$NDVI=\frac{\left(B{\text{8}}_{NIR}-B{\text{4}}_{RED}\right)}{\left(B{\text{8}}_{NIR}+B{\text{4}}_{RED}\right)}$$

where B8 (NIR) corresponds to the near-infrared band and B4 (RED) to the red band of the visible spectrum.

This index is based on the strong contrast between the high reflectance of vegetation in the near-infrared and its strong absorption in the red band, making it a reliable indicator of vegetation vigor.

*NDWI (Normalized Difference Water Index):*


$$NDWI=\frac{\left(B{\text{3}}_{GREEN}-B{\text{8}}_{NIR}\right)}{\left(B{\text{3}}_{GREEN}+B{\text{8}}_{NIR}\right)}$$

where B3 (GREEN) represents the green visible band and B8 (NIR) the near-infrared band.

This index enhances water features by exploiting the difference between the higher reflectance of water in the green band and its strong absorption in the near-infrared region.

For both indices, the value assigned to each buffer corresponded to the zonal mean of all pixel values within the defined area.

Two sets of variables were generated according to their temporal criteria:

NDVI 1 and NDWI 1: obtained from the Sentinel-2A image closest in date to each sandfly and reservoir sampling campaign, reflecting point-in-time environmental conditions around the traps.

NDVI 2 and NDWI 2: obtained from the monthly averages of NDVI and NDWI, integrating all eligible images per month under the cloud cover criteria, representing the average monthly environmental conditions in the surroundings of each sentinel site.

In order to study how environmental variables affect the abundance of sandflies in the sampled traps, variance analysis of the following variables: total phlebotominae abundance, minimum temperature, maximum temperature, minimum relative humidity, maximum relative humidity and win speed was performed. Normality and homoscedasticity assumptions were applied. The analysis considered only traps in which sandflies were found, taking those with p values under 0.05 as significant results.

Data collected during trapping months were analyzed using the Kruskal-Wallis test (5% error) and a post-hoc test. Multivariate analysis was also applied to the data, to obtain the Principal Component Analysis (PCA), which seeks to summarize the variation pattern of the original variables. The software Past 4. 3.00 and Infostat version 2020 were used [53,54]. Additionally, the PCA indicated the percentage of variation explained by each axis [55,56]. Subsequently, differences between sites were evaluated using discriminant analysis (DA).

## 3 Results

### 3.1 Sandfly collection by ecotypes and taxonomic identification

During a 12-month period, a total of 228 traps were placed, with 76 traps in each ecotope (Table 1). Of the traps placed, 64% of the sandfly captures were recorded in the secondary forest, 33% in the primary forest, and 3% in the degraded zone, the latter being the least effective (Table 1). Out of the 30 sites sampled, 24 (80%) recorded sandflies at least once, while 6 sites (20%) had no captures (Table 1). The traps with no captures were in zones B (B1 and B6) and C (C2, C7, C8, and C9) (Table 1).

**Table 1:** Distribution, geolocation, and frequency of CDC light trap placements across ecotopes, and number of sand flies collected per ecological zone in the RBBM, Canindeyú Department, Paraguay, from October 2020 to October 2021.

Light trap codes per zone	Geographical coordinates (UTM)		Vegetation classification by Nadoo and Hill [40]	No. of trap per sites	No. of sandflies collected		
	E (m)	N (m)			Males	Females	Total
A1	648982	7330279	High Forest	7	1	4	5
A2	648930	7330401	Bamboo Forest	7	4	38	42
A3	649035	7330410	Bamboo Forest	8	3	86	89
A4	649282	7330294	Liana Forest	8	8	28	36
A5	649160	7330296	Bamboo Forest	8	25	76	101
A6	649452	7330376	Liana Forest	8	69	44	113
A7	649435	7330507	Bamboo Forest	8	61	56	117
A8	649374	7330231	Liana Forest	8	16	9	25
A9	649359	7330379	Bamboo Forest	7	34	27	61
A10	648901	7330311	Bamboo Forest	7	9	13	22
Subtotal (zone A - Secundary Forest) Relative frecuency 0.64				*76*	*230*	*381*	*611*
B1	648062	7330957	Riparian	7	0	0	0
B2	648060	7331129	Bamboo understory	7	6	2	8
B3	647990	7331251	Bamboo understory	8	6	10	16
B4	648065	7330533	Bamboo understory	8	0	14	14
B5	648133	7330462	High Forest	8	1	6	7
B6	648095	7330745	Bamboo Forest	8	0	0	0
B7	648460	7330335	High Forest	8	16	22	38
B8	648267	7330403	High Forest	8	49	84	133
B9	648183	7330883	Riparian	7	3	1	4
B10	647968	7331406	Bamboo Forest	7	32	67	99
Subtotal (zone B - Primary Forest) Relative frecuency 0.33				*76*	*113*	*206*	*319*
C1	647172	7333608	Meadow	7	1	0	1
C2	647197	7333641	Pasture	7	0	0	0
C3	647180	7333593	Pasture	8	1	2	3
C4	647193	7333566	Pasture	8	0	3	3
C5	647160	7333545	Meadow	8	1	3	4
C6	647123	7333452	Pasture	8	1	0	1
C7	647228	7333539	Degraded	8	0	0	0
C8	647081	7333633	Degraded	8	0	0	0
C9	646911	7333621	Degraded	7	0	0	0
C10	646628	7333567	Grassland	7	8	4	12
Subtotal (zone C - Degraded area) Relative frecuency 0.03				*76*	*12*	*12*	*24*
**Total number**				**228**	**355**	**599**	**954**

In total, 954 sandflies were collected from the three ecotopes, consisting of 599 females and 355 males, representing 12 different species: *Ev. cortelezzii* complex *(n = 340), Brumptomyia brumpti* (n = 312), *Pintomyia monticola* (*n = 161), Psathyromyia lanei (n = 53), Nyssomyia neivai (n = 31), Evandromyia evandroi (n = 19), Brumptomyia avellari (n = 11), Micropygomyia quinquefer (n = 8), Brumptomyia guimaraesi (n = 7), Migonemyia migonei (n = 5), Evandromyia termitophila (n = 4),* and *Psathyromyia shannoni* complex *(n = 3)* (Table 2). The species collected in each of the 30 traps are detailed in Table S1.

**Table 2:** Distribution of phlebotomine sandfly species by ecotope and gender in the RBBM.

No. sandfly species collected	Secundary forest (Zone A)		Primary forest (Zone B)		Degraded area (Zone C)		Total		
	Male	Female	Male	Female	Male	Female	Male	Female	Total
*Ev. cortelezzi* complex	100	120	22	90	4	4	126	214	340
*Brumptomyia brumpti*	84	89	82	42	8	7	174	138	312
*Pintomyia monticola*	8	102	6	44	0	1	14	147	161
*Psathyromyia lanei*	12	34	1	6	0	0	13	40	53
*Nyssomyia neivai*	13	18	0	0	0	0	13	18	31
*Evandromya evandroi*	8	6	1	4	0	0	9	10	19
*Brumptomyia avellari*	0	3	0	8	0	0	0	11	11
*Micropygomyia quinquefer*	0	7	0	1	0	0	0	8	8
*Brumptomyia guimaraesi*	5	0	1	1	0	0	6	1	7
*Migonemyia migonei*	0	2	0	3	0	0	0	5	5
*Evandromyia termitophila*	0	0	0	4	0	0	0	4	4
*Psathyromyia shannoni* complex	0	0	0	3	0	0	0	3	3
*Sub total*	*230*	*381*	*113*	*206*	*12*	*12*	*355*	*599*	*954*
**Total number (%)**	**611 (64)**		**319 (33)**		**24 (3)**		**(37)**	**(63)**	**(100)**

Regarding the sex distribution of the collected specimens, a predominance of females was observed in all the species collected, except in *Br. guimaraesi* and *Br. brumpti*, where a higher number of males were recorded (Table 2).

When analyzing each studied ecotope, it was observed that in the secondary forest, 230 males and 381 females were collected, totaling 611 specimens (64%) from 10 different species (Tables 1 and 2). The most abundant species in this zone were *Ev. cortelezzii* complex, followed by *Br. brumpti*, *Pi. monticola*, *Pa. lanei*, and *Ny. neivai*. Meanwhile, species such as *Ev. evandroi*, *M. quinquefer*, *Br. guimaraesi*, *Br. avellari*, and *Mg. migonei* were captured in smaller quantities (Table 2). All traps in this zone collected specimens at least once, with traps in sites A5, A6, and A7 being the most effective, capturing 111, 113, and 117 sandflies, respectively, while trap A1 was the least effective, with only 5 specimens captured (Table 1). Notably, *Ny. neivai* was captured exclusively in this zone, and only male specimens of *Br. guimaraesi* and female specimens of *Br. avellari* and *M. quinquefer* were collected (Table 2). There were no captures of *Ev. termitophila* and *Pa. shannoni* complex in this zone.

In the primary forest ecotope, 113 males and 206 females were collected, totaling 319 specimens (33%) from 11 different species (Tables 1 and 2). *Br. brumpti* was the most abundant species, followed by *Ev. cortelezzii* complex and *Pi. monticola*, while species such as *Pa. lanei*, *Ev. evandroi*, *Br. avellari*, *M. quinquefer*, *Br. guimaraesi*, *Mg. migonei*, *Ev. termitophila*, and *Pa. shannoni* complex were captured in smaller quantities (Table 2). Traps B8 and B10, which captured 33 and 99 sandflies, respectively, were the most effective compared to the rest, in contrast to traps B1 and B6, where no captures were recorded (Table 1). Notably, *Ev. termitophila* and *Pa. shannoni* complex were captured exclusively in this zone (Table 2). Regarding the species captured in low quantities, almost all were females, except for *Br. guimaraesi* and *Ev. evandroi*, where one male of each species was also collected (Table 2).

In the degraded zone ecotope, 12 males and 12 females were collected, totaling 24 specimens (3%) from 3 different species (Tables 1 and 2). The predominant species was *Br. brumpti*, followed by *Ev. cortelezzii* complex, and a single female specimen of *Pi. monticola* (Table 2), which were also present in zones A and B. Captures in this zone were limited to only 6 traps, with trap C10 being the most productive, capturing 12 specimens, while traps C2, C7, C8, and C9 recorded no captures (Table 1).

### 3.2 Seasonality

**Fig 2:**
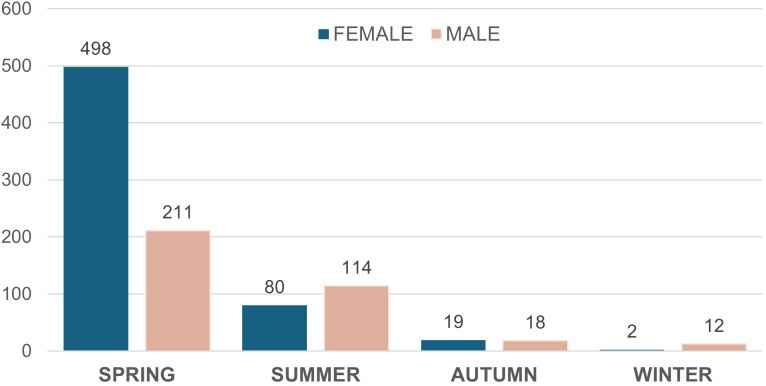
Number and sex of Phlebotomine sandflies captured by season in the RBBM in the period 2020-2021.

The highest number of sandflies captures occurred in the spring, with 709 specimens collected, of which 70% were females. In contrast, during the summer, 194 specimens were captured, with males being more abundant, representing 59%. Sandfly captures decreased considerably in autumn and winter. In autumn, 37 specimens were recorded, with similar proportions of males and females, while in winter, with only 14 specimens, males represented 86% of the captures (Fig 2).

The diversity of sandfly species showed variation throughout the year, with 11 species collected in spring, 8 in summer, 6 in autumn, and 5 in winter (Table 3). The species *Br. brumpti*, *Ev. cortelezzii* complex, *Ev. evandroi*, and *Br. avellari* were present during all seasons, while *Br. guimaraesi*, *Ev. termitophila*, and *Pa. shannoni* complex were only detected in spring, and *Ny. neivai* was only present in summer (Table 3).

**Table 3:** Diversity and abundance of phlebotomine sand flies species by season in the RBBM.

Sandfly species	Seasons			
	Spring	Summer	Autumn	Winter
*Ev. cortelezzi* complex	251	74	8	7
*Brumptomyia brumpti*	245	50	14	3
*Evandromyia evandroi*	7	5	5	2
*Brumptomyia avellari*	3	1	6	1
*Pintomyia monticola*	154	6	0	1
*Psathyromyia lanei*	25	26	2	0
*Nyssomyia neivai*	0	31	0	0
*Migonemyia migonei*	4	1	0	0
*Micropygomyia quinquefer*	6	0	2	0
*Brumptomyia guimaraesi*	7	0	0	0
*Evandromyia termitophila*	4	0	0	0
*Psathyromyia shannoni* complex	3	0	0	0

### 3.3 Molecular detection of *Leishmania* DNA in Phlebotominae

To determine the presence of *Leishmania* spp. DNA in sandflies, 599 females were processed individually. Of these, 69 specimens from 9 different species tested positive (11.5%) (Table 4). The species with the highest positivity rates were *Ev. cortelezzii* complex (20.3%), *Pi. monticola* (18.8%), and *Br. brumpti* (17.4%), which were also the most captured, followed by *Br. avellari* (15.9%) and *Pa. lanei* (11.6%). Species like *Ev. evandroi*, *M. quinquefer*, *Ny. neivai*, and *Mg. migonei* showed positivity rates of 4.3% or lower (Table 4). A significant finding was that all females of *Br. avellari* were positive. Species that tested negative for *Leishmania* spp. DNA were *Pa. shannoni* complex, *Br. guimaraesi*, and *Ev. termitophila*.

**Table 4:** Number and percentage of female sandfly species infected with *Leishmania* spp. in the RBBM.

Sandfly species	No. female sandfly captured (%)	No. sandfly with *Leishmania* spp. (%)	No. sandfly with *Leishmania* spp. (%)	
			*L. amazonensis*	*L. infantum*
*Ev. cortelezzi* complex	214 (35.7)	14 (20.3)	9 (64.3)	5 (35.7)
*Pintomyia monticola*	147 (24.5)	13 (18.8)	11 (84.6)	2 (15.4)
*Brumptomyia brumpti*	138 (23.0)	12 (17.4)	10 (83.3)	2 (16.7)
*Psathyromyia lanei*	40 (6.7)	8 (11.6)	5 (62.5)	3 (37.5)
*Brumptomyia avellari*	11 (1.8)	11 (15.9)	5 (45.5)	6 (54.5)
*Evandromya evandroi*	10 (1.7)	3 (4.3)	0	3 (100)
*Nyssomyia neivai*	18 (3.0)	3 (4.3)	3 (100)	0
*Micropygomyia quinquefer*	8 (1.3)	3 (4.3)	1 (33.3)	2 (66.7)
*Migonemia migonei*	5 (0.8)	2 (2.9)	1 (50)	1 (50)
*Psathyromyia shannoni* complex	3 (0.5)	0	0	0
*Brumptomyia guimaraesi*	1 (0.2)	0	0	0
*Evandromyia termitophila*	4 (0.7)	0	0	0
**Total No. (%)**	**599 (100)**	**69 (11.5)**	**45 (65.2)**	**24 (34.8)**

The *Leishmania* spp. species circulating in the study areas were *L. amazonensis* (65.2%) and *L. infantum* (34.8%) (Table 4). Species from the *Ev. cortelezzii* complex, *Pi. monticola*, *Br. brumpti*, *Pa lanei*, *Br. avellari*, *Mg. quinquefer*, and *Mg. migonei* carried either *L. amazonensis* or *L. infantum* DNA (Table 3). In *Ev. evandroi*, only *L. infantum* DNA was detected, while in *Ny. neivai*, only *L. amazonensis* DNA was identified (Table 4). No sandfly tested positive for both species simultaneously.

### 3.4 *Leishmania* spp. DNA presence in collection sites and seasonality

Regarding the collection sites of the 69 specimens positive for *Leishmania* spp., 48 were collected from the secondary forest (69.6%), 20 from the primary forest (29.0%), and 1 from the degraded area (1.4%) (Table 4). In the secondary forest, 9 positive species were detected, 6 in the primary forest, and 1 in the degraded area (Table 5). It should be highlighted that in the secondary forest, *L. amazonensis* predominated, while in the primary forest, the circulation of *L. amazonensis* and *L. infantum* was similar. In the only positive specimen captured in the degraded area, *L. amazonensis* DNA was detected.

**Table 5:** Number and infection percentages of female sandfly species with *L. amazonensis* and *L. infantum* in different ecotopes.

Sandfly species	*Leishmania* species captured by zone					
	Secundary forest (Zone A)		Primary forest (Zone B)		Degraded area (Zone C)	
	*L.* *amazonensis*	*L.* *infantum*	*L. amazonensis*	*L. infantum*	*L. amazonensis*	*L. infantum*
*Brumptomyia brumpti*	7	2	2	0	1	0
*Brumptomyia avellari*	2	1	3	6	0	0
*Ev. cortelezzi* complex	6	5	3	0	0	0
*Evandromya evandroi*	0	1	0	2	0	0
*Micropygomyia quinquefer*	0	2	1	0	0	0
*Pintomyia monticola*	9	1	2	1	0	0
*Nyssomyia neivai*	3	0	0	0	0	0
*Migonemyia migonei*	1	1	0	0	0	0
*Psathyromyia lanei*	5	2	0	0	0	0
**Total number**	**33**	**15**	**11**	**9**	**1**	**0**
**Percentage per zone (%)**	**68.8**	**31.2**	**55%**	**45%**	**100%**	**0%**
**Total Percentage (%)**	**69.6**		**29%**		**1.4%**	

Sequencing analysis of the amplified ITS1 fragments revealed that 45 (65.2%) of the *L. amazonensis-*positive specimens had an identity percentage between 98.84% and 99.22%, while the 24 (34.8%) *L. infantum*-positive specimens had an identity percentage between 99.17% and 100% (Tables 4 and 6).

**Table 6:** Abundance and ecological distribution of sandfly species positive for *L. infantum* and *L. amazonensis* identified by sequencing.

Sandfly species	Number of sandflies species per zone	Zone	*Leishmania* species detected by ITS-1	Closest match in GeneBank accession number	Identity (%)
*Brumptomyia avellari*	1	Secundary forest	*L. infantum*	MN503527.1	99.17
	6	Primary forest	*L. infantum*	MN503527.1	99.17
	2	Secundary forest	*L. amazonensis*	AF339753.1	99.22
	3	Primary forest	*L. amazonensis*	AF339753.1	99.22
*Brumptomyia brumpti*	7	Secundary forest	*L. amazonensis*	AF339753.1	99.22
	2	Primary forest	*L. amazonensis*	AF339753.1	99.22
	1	Degraded area	*L. amazonensis*	AF339753.1	99.22
	2	Secundary forest	*L. infantum*	MN503527.1	100
*Pintomyia monticola*	9	Secundary forest	*L. amazonensis*	AF339753.1	99.22
	2	Primary forest	*L. amazonensis*	AF339753.1	99.22
	1	Secundary forest	*L. infantum*	MN503527.1	100
	1	Primary forest	*L. infantum*	AF339753.1	99.22
*Ev. cortelezzi* complex	6	Secundary forest	*L. amazonensis*	AF339753.1	99.22
	3	Primary forest	*L. amazonensis*	AF339753.1	99.22
	5	Secundary forest	*L. infantum*	MN503527.1	100
*Psathyromyia lanei*	5	Secundary forest	*L. amazonensis*	AF339753.1	99.22
	2	Secundary forest	*L. infantum*	MN503527.1	100
*Micropygomyia quinquefer*	2	Secundary forest	*L. infantum*	MN503527.1	100
	1	Primary forest	*L. amazonensis*	AF339753.1	99.22
*Migonemyia migonei*	1	Secundary forest	*L. infantum*	MN503527.1	100
	1	Secundary forest	*L. amazonensis*	AF339753.1	99.22
*Evandromyia evandroi*	1	Secundary forest	*L. infantum*	MN503527.1	99.17
	2	Primary forest	*L. infantum*	MN503527.1	99.17
*Nyssomyia neivai*	3	Secundary forest	*L. amazonensis*	AF339753.1	98.84

**Fig 3:**
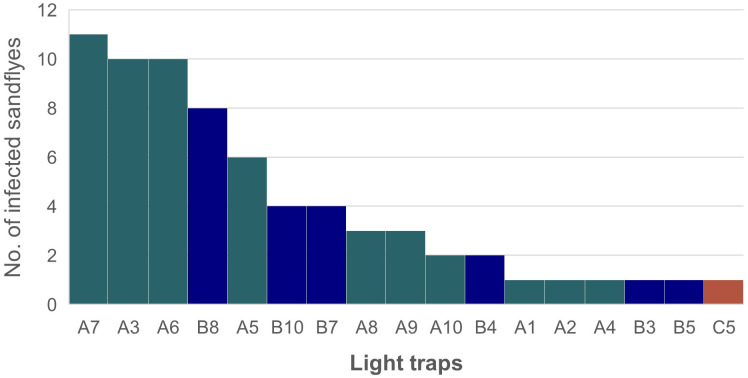
Abundance of sandflies positive to *Leishmania* spp. distributed throughout traps and collection area.

**Fig 4:**
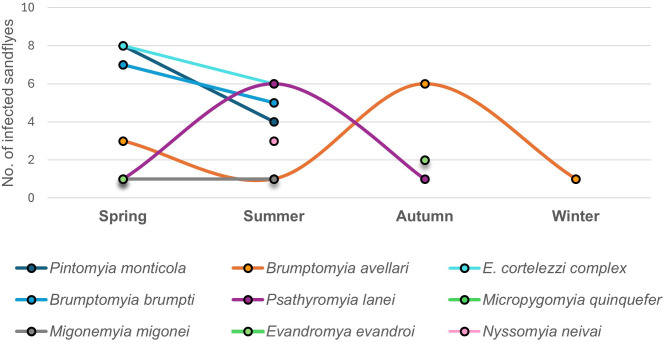
Diversity and abundance of *Leishmania* spp. positive sandflies species captured by season.

Out of the 30 sites where light traps were placed, 17 of them captured sandflies positive for *Leishmania* spp. (Fig 3). In all the traps placed in the secondary forest, positive specimens were captured, occasionally with higher abundance in traps A7, A3, A6, and A5 (Fig 4). In the primary forest, 6 out of 10 traps captured positive specimens, with traps B8, B7, and B10 showing the highest abundance (Fig 3). In the degraded area, only trap C5 captured a sandfly positive for *L. amazonensis* (Fig 3).

**Fig 5:**
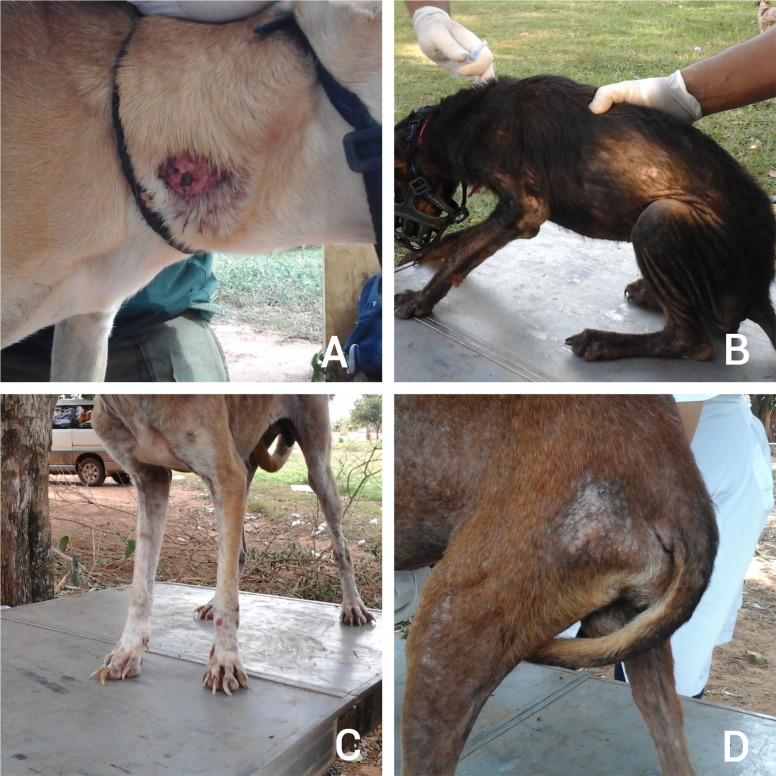
Clinical signs associated with Leishmaniasis in dogs. A. Skyn ulcer B. Alopecia C. Nail overgrowth D. Hair loss and skin ulcers.

The greatest diversity of species positive for *Leishmania* spp. was observed in spring and summer, with 8 and 7 species registered, respectively (Fig 4). In contrast, autumn and winter showed lower diversity, with only 4 and 2 positive species recorded in each season, respectively (Fig 5). Notably, *Br. avellari* was the only species detected as positive for *Leishmania* spp. in all seasons of the year, followed by *Pi. monticola*, which was detected in spring, summer, and autumn (Fig 4).

### 3.5 rK39 rapid diagnostic test for canine visceral leishmaniasis and molecular detection of Leishmania DNA in ectoparasites

A parallel study was conducted in 40 mixed-breed dogs (15 females and 25 males) belonging to 26 households in the Arroyo Bandera indigenous community (Table 7).

**Table 7:** Demographic characteristics and infection by *Leishmania* spp. of dogs and their ectoparasites of the Arroyo Bandera Indigenous Community.

No. House	No. Dog	Sex	Age (years)	Antileishmania Inmunological test results	Dogs Ectoparasites colected (n)		*Leishmania* PCR test results	
				rk39 test in dogs	*Ctenocephalides* spp.	*Rhipicephalus* spp.	*Ctenocephalides* spp.	*Rhipicephalus* spp.
1	1	Male	2	Negative	2	0	*L. amazonensis*	–
2	2	Male	3	Negative	0	0	–	–
3	3	Male	1	Negative	3	0	*L. amazonensis*	–
4	4	Female	1	Negative	0	2	–	Negative
5	5	Female	2	Negative	2	0	Negative	–
6	6	Male	2	Negative	2	0	Negative	–
	7	Female	2	Negative	3	0	*L. amazonensis*	–
	8	Female	5	Negative	3	0	Negative	–
7	9	Male	8	Negative	3	0	*L. amazonensis*	–
	10	Male	5	Negative	0	0	–	–
8	11	Male	2	Negative	2	0	*L. amazonensis*	–
9	12	Female	6	Negative	0	0	–	–
10	13	Female	1	Negative	1	0	*L. amazonensis*	–
11	14	Male	1	Negative	2	0	*L. amazonensis*	–
	15	Female	2	Negative	2	0	Negative	–
12	16	Male	1	Negative	2	0	*L. amazonensis*	–
	17	Male	1	Negative	2	0	*L. amazonensis*	–
	18	Male	2	Negative	2	0	*L. amazonensis*	–
	19	Male	2	Negative	2	0	*L. amazonensis*	–
13	20	Male	7	Negative	3	0	Negative	–
	21	Male	0.9	Negative	3	0	Negative	–
	22	Male	9	Negative	3	0	Negative	–
14	23	Female	8	Negative	0	1	–	Negative
15	24	Macho	8	Negative	3	0	Negative	–
	25	Female	1	Negative	3	1	Negative	Negative
	26	Male	7	Negative	2	0	Negative	–
16	27	Male	1	Negative	3	0	*L. amazonensis*	–
17	28	Male	1	Negative	3	0	*L. amazonensis*	–
18	29	Female	4	Negative	3	0	*L. amazonensis*	–
19	30	Female	1	Negative	3	0	*L. amazonensis*	–
20	31	Male	0.1	Negative	3	0	Negative	–
21	32	Female	3	Negative	1	2	Negative	*L. amazonensis*
	33	Female	4	Negative	3	0	Negative	–
22	34	Male	1	Negative	1	0	Negative	–
23	35	Male	4	Negative	3	0	*L. amazonensis*	–
24	36	Male	3	Negative	0	0	–	–
25	37	Female	1	Negative	3	0	*L. amazonensis*	–
	38	Female	4	Negative	0	0	–	–
	39	Male	2	Negative	1	0	*L. amazonensis*	–
26	40	Male	1	Negative	4	0	Negative	–

**Fig 6:**
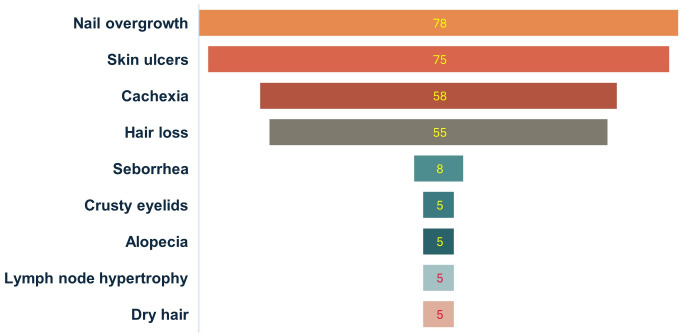
Percentage of the variety of clinical signs associated with leishmaniasis in dogs from the indigenous community of Arroyo Bandera.

The dogs underwent an immunological analysis using the rapid immunochromatographic rK39 test, which detects antibodies against the *Leishmania donovani* species complex. All dogs tested negative for the rK39 test (Table 7), however, all displayed clinical signs associated with leishmaniasis (Fig 5), such as overgrowth of nails (78%), skin ulcers on ears, face, or body (75%), cachexia (58%), hair loss (55%), seborrhea (8%), eye discharge (5%), alopecia (5%), lymph node hypertrophy (5%), and dry hair (5%) (Fig 6).

Additionally, ectoparasites were collected from 37 of the 40 dogs (92.5%), which were analyzed for the presence of *Leishmania* spp. using molecular techniques. In total, 81 fleas of the *Ctenocephalides* genus were collected from 33 dogs (82.5%), and 6 ticks of the *Rhipicephalus* genus were collected from 4 dogs (12.5%) (Table 7).

Fleas collected from each dog were processed individually or in groups, totaling 33 samples, of which 18 (54.5%) were positive for *Leishmania* spp. Ticks were processed individually, with DNA from *Leishmania* spp. detected in only one specimen. All positive ectoparasites were identified by sequencing as *L. amazonensis* (Table 7).

### 3.6 Meso-scale environmental variables and sandfly abundance

The subsequent correlation analysis between the aforementioned mesoscale environmental variables and the abundance of sandflies revealed significant patterns, thereby facilitating a more profound comprehension of the environmental factors that influence the distribution of these vectors. Among the climatic variables, mean temperature exhibited a modest yet statistically significant positive correlation (r = 0.235; *p* < 0.001), suggesting that moderately high temperatures may favor the activity and abundance of sandflies. Conversely, relative humidity, maximum humidity, and wind speed exhibited no statistically significant correlations. However, minimum humidity exhibited a modest positive correlation (r = 0.179; *p* = 0.0032), suggesting that low levels of nocturnal humidity might still permit the activity of these insects under specific conditions (S2 Table).

Regarding the relationship between vegetation and humidity indices and the abundance of sandflies, the NDVI exhibited a significant positive correlation at the three evaluated scales (25, 50, and 100 m), with consistent values around r ≈ 0.29 and very high levels of significance (*p* < 0.001) (S2 Table). These findings corroborate the hypothesis that areas with higher vegetation density are associated with a higher presence of sandflies, likely due to the greater availability of shelter, moisture, and favorable microclimatic conditions. Conversely, NDWI exhibited a negative correlation across all scales (r ≈ -0.20; *p* < 0.01), suggesting that the lower moisture content in surface vegetation might be associated with higher sandfly populations (S2 Table).

In terms of land cover, a negative correlation was observed with agricultural and bare soil areas, particularly at distances of 50 and 100 meters from the sentinel site (r = -0.29 to -0.18; *p* < 0.01) (S2 Table). These negative associations suggest that more disturbed landscapes reduce the presence of sandflies, possibly due to loss of suitable habitat and microenvironmental alterations. In contrast, forest cover exhibited a significant positive correlation (r = 0.26 to 0.259; *p* < 0.001) at 50 and 100 meters (S2 Table), thereby reinforcing the hypothesis that forested environments, both primary and secondary, provide optimal conditions for the survival and reproduction of sandflies.

Finally, no statistically significant relationships were identified between proximity to water and abundance of sandflies in any of the evaluated scales. These findings suggest that, at least within the context of this particular ecological study, the presence of water does not significantly influence the population density of these insects (S2 Table).

**Fig 7:**
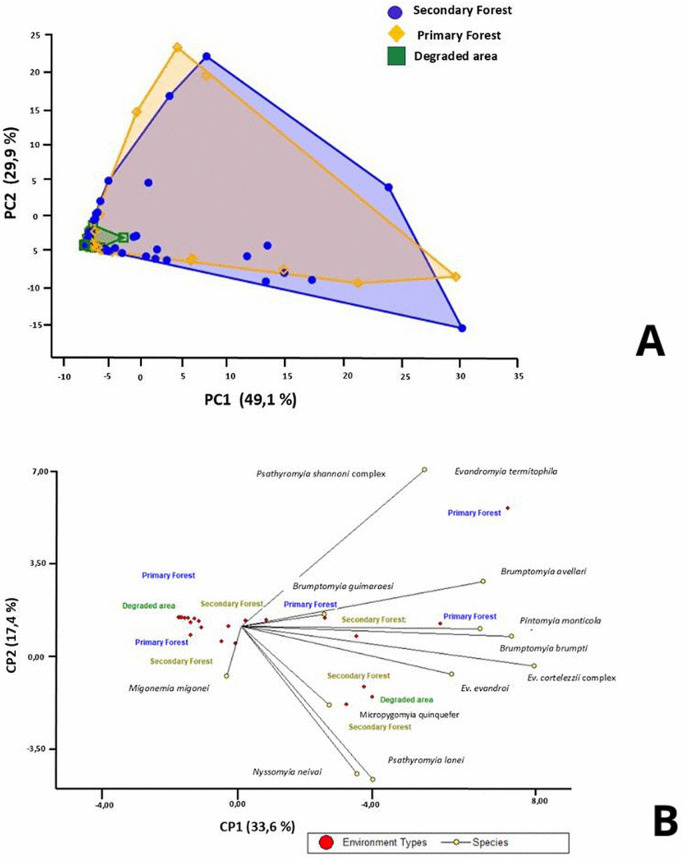
Principal component analysis (PCA), according to (A) Number of sandfly species per capture site (B) Total number of male and female of the species captured and the type of environment.

Principal component analysis (PCA) revealed clear differentiations between the evaluated types of environments in terms of sandfly species composition. The first principal component (PC1) explained 49% of the observed variability, while the second component (PC2) accounted for 29.9%, indicating a high discrimination capacity between sites (Fig 7A).

**Fig 8:**
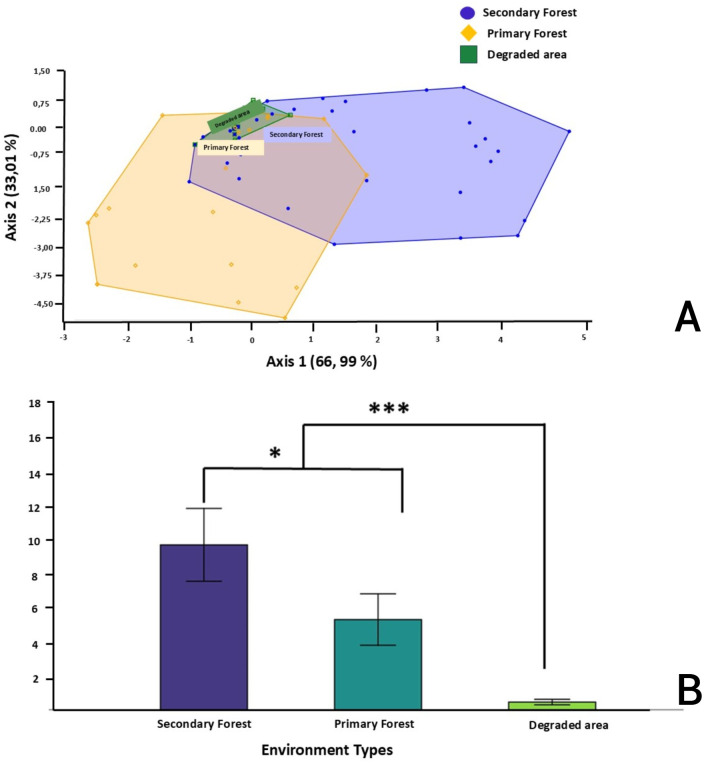
Discriminant analysis of (A) capture sites (B) Sandfly abundance and their significant differences *(P < 0.05) and ***(P < 0.00) according to capture sites.

Within this distribution, the secondary forest exhibited the most extensive dispersion of points in the multivariate space, suggesting a greater degree of diversity and heterogeneity in species composition. In contrast, the primary forest exhibited intermediate variability, while degraded areas demonstrated more pronounced clustering, indicating lower variability and more homogeneous communities (Fig 8A).

The secondary forest exhibited the highest species richness and abundance, including *Ev. cortelezzii* complex, *Pi. monticola, Br. brumpti, M. quinquefer, Mg. migonei, Ny. neivai, Br. guimaraesi,* and *Pa. lanei.* Conversely, the primary forest exhibited a distinct biodiversity, featuring species such as *Pa. shannoni* complex, *Pa. lanei, Ev. termitophila* and *Mg. migonei*. In contrast, degraded areas showed lower specific richness, with a predominance of *Mg. migonei* and *M. quinquefer* (Fig 7B).

Discriminant analysis (DA) confirmed significant differences in the different species composition and abundance of sandflies between habitat types (Fig 8A). While primary and secondary forests exhibited some species overlap, the secondary forest demonstrated a higher density of sandflies. A statistically significant increase in total abundance was observed in the secondary forest compared to the primary forest (*p* < 0.05) and degraded areas (*p* < 0.001), which recorded the lowest number of sandflies (Fig 8B). The findings indicate that secondary forests offer particularly favorable ecological conditions for the proliferation of sandflies, both in terms of diversity and density.

## 4 Discussion

This is the first study exploring the abundance and presence of *Leishmania* DNA in sandflies in the RBBM, a protected area in the Atlantic Forest (Bosque Atlántico) of Alto Paraná, Paraguay, near the border with Brazil. We selected the RBBM as our study area because it offers a unique and representative environment of the Atlantic Forest (Bosque Atlántico) biodiversity and the transitional ecosystem with the Cerrado. As the largest and most protected remnant of this biome, the RBBM provides a relatively intact habitat where wildlife can be studied as natural reservoirs of pathogens, which is vital for understanding the dynamics of zoonotic disease transmission [57]. The reserve is a critical area for monitoring and researching vector-borne pathogens due to its high biodiversity, conservation of natural habitats, and the presence of numerous vertebrate species acting as reservoirs. These characteristics make the RBBM a strategic site to advance the understanding of the ecology and transmission dynamics of zoonotic diseases in Paraguay and the region.

The capture of sandflies using CDC mini light traps allowed us to sample various ecotopes and proved highly efficient, providing accurate data on the species richness and diversity of sandflies present, as observed in other studies [58].

The highest abundance and diversity of sandflies were recorded in the primary and secondary forests of RBBM, forest areas with less anthropogenic impact compared to the degraded area (farm). These less disturbed areas act as ecological refuges, maintaining a higher species distribution richness [59]. This trend aligns with studies in southern Brazil, where a clear correlation was found between insect diversity and the presence of remnant forests [60]. These findings emphasize the importance of conserving these ecosystems, as their protection is a pillar in maintaining the biological diversity of sandflies and other organisms coexisting in these habitats.

Studies aimed at identifying molecular targets for the accurate diagnosis of leishmaniasis have shown that the ITS-1 region is highly effective in detecting all *Leishmania* species, supporting its choice as a marker in this study [61]. Studies on natural infection rates have revealed that PCR is more sensitive and specific than dissection for demonstrating the presence of *Leishmania* in sandflies [62–64]. In our study, amplification of the ITS1 fragment was successful in 69 females, distributed across nine species captured in the three ecotopes studied. The largest number of sandflies was captured in spring and summer, periods known for intense sandfly activity, as temperature and humidity conditions during these months favor their proliferation and the transmission of *Leishmania* spp. [65,66]. The study of potential vectors is relevant to leishmaniasis epidemiology, especially in regions where specific vectors are absent. These vectors may occasionally intervene in parasite transmission, allowing their persistence and favoring the expansion of their geographic distribution to non-endemic areas [67–72].

This study observed a high diversity of sandflies, with a total of 12 different species identified, 10 of which are reported for the first time in the Department of Canindeyú (Paraguay), highlighting the importance of this forest area and the impressive biodiversity of the region, which had not been previously sampled.

The *Ev. cortelezzii* complex is distributed across Argentina, Bolivia, Brazil, Paraguay, Peru, and Uruguay [73]. In Paraguay, it has been recorded in the departments of Alto Paraná, Itapúa, Cordillera, Central, and Boquerón [6]. This study marks the first record of the species in Canindeyú, with specimens captured in all three ecotopes studied. In Argentina, it inhabits a variety of habitats, including tree trunks and hollows, roots, marginal areas, peridomestic constructions with animals, and both external and internal walls of human dwellings [68]. In this study, we found specimens with *L. infantum* DNA in secondary forests and *L. amazonensis* in primary and secondary forests. Natural infections of this species with *L. braziliensis* have been reported in Argentina’s Chaco province, reinforcing the hypothesis of its potential role in transmitting ATL [74]. In Brazil, cases of specimens infected with *L. infantum* [75–77] and *L. braziliensis* [78] have been documented, suggesting its potential role in the transmission cycles of tegumentary and visceral leishmaniasis in wild or rural environments [79]. This study documents for the first time the presence of *L. amazonensis* DNA in *Ev. cortelezzii* complex, observed in specimens collected from primary and secondary forests. The detection of *L. amazonensis* DNA suggests its potential involvement in the transmission cycles of cutaneous and mucocutaneous leishmaniasis and highlights the need for further studies to confirm its vector competence and ecological role in the region.

*Pi. monticola* is distributed in Argentina, Brazil, and Peru [73]. In Paraguay, it has been recorded in the departments of San Pedro and Caaguazú [68], and this study represents the first report of its presence in Canindeyú. In Brazil, it has been captured in areas of fragmented and closed native forests, showing a notable preference for CDC light traps [80,81]. Regarding the presence of *Leishmania* spp., in Minas Gerais, Brazil, specimens with *L. braziliensis* have been reported in rural and forested areas [82], while protected areas have recorded specimens with *L. infantum* DNA [83]. In Argentina, this sandfly species has been recorded in an endemic area of ATL in the Corrientes province, though no infections by *Leishmania* spp. have been reported [84]. Our study is pioneering in documenting *Pi. monticola* with *L. amazonensis* and *L. infantum* in primary and secondary forests, marking the first global report of the presence of *L. amazonensis* DNA in this species. Additionally, *P. monticola* was the species with the highest number of specimens positive for *L. amazonensis*, most of which were captured in the secondary forest area, where there is high human circulation due to the ecotourism and education zone of the RBBM. This finding is particularly relevant, as previous studies have indicated that *Pi. monticola* is an essentially forest-dwelling, highly anthropophilic species susceptible to infections by *Leishmania* spp. [85–87].

*Brumptomya* species occur in the Atlantic Forest (Bosque Atlántico) area and primarily inhabit wild environments. Their presence is considered an indicator of environmental health [88], although recent habitats also include domestic animal annexes and marginal areas [89]. Unfortunately, perhaps due to its lack of epidemiological importance and low or absent anthropophily [90], this genus has received limited attention. Nevertheless, recent reports of molecular detection of *Leishmania* DNA in species of this genus have been recorded in Ecuador [91], Mexico [92] and Brazil [60,58,93]. These detections raise questions about the feeding habits of this genus, which is thought to feed exclusively on the blood of armadillos (Dasypodidae), or about the possible role of armadillos as reservoirs of *Leishmania* spp. in wild transmission cycles. Information on infections in *Brumptomyia* species with *Leishmania* spp. is still limited. Documented cases so far come from Acre, Brazil, where specimens infected with *L. braziliensis* [59] and *Leishmania* spp. [58] have been found; from *Br. leopoldoi* in Ecuador [91]; from *Br. mesai* in Mexico [92]; and from *Br. nitzulescui* in Minas Gerais, Brazil [93]. In this study, we collected several species of this genus, whose findings are described below.

*Br. brumpti* is found in Argentina, Bolivia, Brazil, and Paraguay, where it has been captured in the departments of Amambay, San Pedro, Canindeyú, Alto Paraná, Cordillera, Paraguarí, Caazapá, and Itapúa in intra, peri, and extra-domiciliary environments [6]. This species occupies a wide range of habitats, from armadillo shelters and tree trunks to caves, cracks, rural areas, and recently deforested zones [68]. In our study, we detected *Leishmania* spp. DNA in all three ecotopes studied. Notably, *Br. brumpti* was the only species positive for *L. amazonensis* collected in the farm zone. In specimens captured in the secondary forest, we detected *L. infantum* and *L. amazonensis*, while in the primary forest, only *L. amazonensis* was circulating.

*Br. avellari***,** distributed in Argentina, Bolivia, Brazil, Colombia, Panama, Peru, and Venezuela [73], has been recorded in Paraguay in the departments of Concepción, San Pedro, Cordillera, Caaguazú, Ñeembucú, Itapúa, and Misiones, inhabiting intra- and peridomiciliary areas [6]. In this study, we report for the first time its presence in Canindeyú in primary and secondary forest and with the presence of DNA of *L. amazonensis* and *L. infantum*.

*Br. guimaraesi***,** distributed in Argentina, Brazil, and Paraguay, has been previously recorded in the departments of San Pedro, Misiones, and Itapúa [6]. This study represents the first finding of the species in the department of Canindeyú. In Brazil, it has been found in fragmented native forest areas, while in Argentina, it was found in the urban environment of Clorinda, a border town with Paraguay [81]. During our research, we captured specimens in primary and secondary forests; however, none tested positive for *Leishmania* spp.

*Pa. lanei* is found in Brazil, Argentina, and Paraguay [73], with previous records in San Pedro and Caaguazú [68]. Our study marks the first finding of this species in Canindeyú. This sandfly is found in various environments, from domiciles and peridomiciles to chicken coops, pigsties, and in tree hollows and canopies. In Argentina, it has been reported in Misiones [94], while in Brazil, it has been found in a protected park dedicated to ecotourism in the state of Alagoas [95], as in the Parque Estadual do Alto Ribeira (PETAR), located within an Atlantic Forest reserve, an important ecotourism attraction in the Ribeira Valley and an endemic area for ATL [85]. In our study, we captured specimens in both primary and secondary forests, where several specimens tested positive for *L. infantum* or *L. amazonensis*, all linked to the secondary forest. This study represents the first report of *Pa. lanei* with DNA of *L. infantum* and *L. amazonensis*.

*Ev. evandroi* has been observed in both semi-arid regions [96] and conservation areas with high anthropogenic activity [97]. In northeastern Argentina, it has been captured in peridomiciliary peri-urban areas [68]. A study conducted in northeastern Brazil identified specimens with *L. amazonensis* DNA [98]. In this study, we identified several specimens with DNA of *L. infantum* exclusively, from both the primary and secondary forests. This discovery is especially relevant given the anthropophilic behavior of *Ev. evandroi* and its association with peridomiciliary environments, where it feeds on a wide variety of animal species [99].

*Ny. neivai* is found in Argentina, Bolivia, Brazil, and is widely known in Paraguay, where it occurs throughout the eastern region, except for Ñeembucú, and in the department of Presidente Hayes in the western region [6]. This species adapts to various environments, including urban, rural, periurban, wild, and secondary vegetation areas [100]. In Argentina and Brazil, it is the main vector of ATL caused by *L. braziliensis* [100,101] and the presence of *L. infantum* DNA has been documented in different states of Brazil [76,102,103]. In Paraguay, this species is associated with the transmission of *L. braziliensis* in the Central department, according to epidemiological evidence [10]. In our study, we report for the first time *Ny. neivai* with the presence of DNA of *L. amazonensis* captured in a secondary forest. It is worth noting that *Ny. neivai* can disperse distances between 250–520 meters across pastures or peridomiciliary areas, showing its ability to spread in open areas [104], facilitated by the degradation of primary environments.

*Mi. quinquefer* has been recorded in Argentina, Bolivia, and Brazil [73] and was recently identified in Paraguay, with captures in intradomiciliary environments in San Pedro, Misiones, and Itapúa [6]. This study marks the first record of its presence in Canindeyú. In this study, several specimens were captured in secondary forests, positive for *L*. *infantum* DNA, and in primary forests, positive for L. *amazonensis* DNA. These findings are consistent with observations in other regions, such as in Puerto Iguazú, Argentina, where *L. infantum* DNA was detected [98], and in northeastern Brazil, where *L. amazonensis* was found [105].

*Mg. migonei* is a sandfly species with a distribution across much of South America, including Argentina, Bolivia, Brazil, Colombia, Paraguay, Peru, Trinidad and Tobago, and Venezuela [73]. In Paraguay, it has been documented in urban areas such as Asunción and in border regions with Brazil, like Amambay and Canindeyú, highlighting its adaptability to various environments [6]. Historically, *Mg. migonei* has been considered a species typically found in dense primary forests, although it can also be found in less dense vegetation areas. While primarily associated with forested environments, it is not uncommon to find it in indoor areas and animal shelters, reflecting its anthropophilic behavior [106–109]. This adaptability may explain its role as a vector of zoonotic diseases in urban and peri-urban areas. In our study, we captured specimens of *Mg. migonei* in primary and secondary forests. Specimens positive for *L. infantum* and *L. amazonensis* DNA were found in secondary forests, suggesting that these areas, despite being less dense and more disturbed by human activity, may serve as key habitats for the transmission of *Leishmania* spp. This finding is particularly relevant in the epidemiological context, as *Mg. migonei* has been implicated as a vector of *L. braziliensis* in Paraguay [10] and Brazil [110]. Additionally, previous studies in Santiago del Estero, Argentina, and the states of Rio de Janeiro and Pernambuco, Brazil, have suggested that *Mg. migonei* could be a potential vector of *L. infantum*, given that natural infections have been identified in these regions [111–113]. An experimental study has also shown that *L. amazonensis* can develop in this species [114], further reinforcing its significance in transmitting different species of *Leishmania*. Due to the epidemiological importance of this species, it is worth noting that this study is the first report of *Mg. migonei* with *L. amazonensis* DNA and the first record for Paraguay of its positivity to both *L. infantum* and *L. amazonensis*.

In the *Pa. shannoni* complex, the species *Pa. shannoni* is widely distributed across the Americas, with documented presence in Belize, Bolivia, Colombia, Costa Rica, French Guiana, Guatemala, Honduras, Mexico, Nicaragua, Panama, Peru, Suriname, Trinidad and Tobago, the United States, and Venezuela [73]. This species shows a notable preference for anthropogenic environments, frequently captured in intra- and peridomiciliary areas [115]. In Paraguay, it is common in the eastern region of the country and has been documented in nearly all departments except Canindeyú, Ñeembucú, and Misiones [6]. Therefore, with the findings of this study, the presence of *Pa. shannoni* complex in Canindeyú is documented for the first time. Regional studies have revealed the high susceptibility of this species to infection with *L. chagasi/infantum* in Peru, where its behavior is comparable to that of *Lu. longipalpis* [116]. Additionally, it has been documented that this species can present natural infections with *Trypanosoma rangeli* and has been identified as a vector of *L. chagasi* in Venezuela [117]. Laboratory studies have shown it is susceptible to *L. chagasi* and *L. panamensis*, and it is considered a competent vector for *L. mexicana*. This transmission capability, particularly in the United States, reinforces its status as a potential vector of leishmaniasis, especially *L. braziliensis* [118–120]. However, during our investigation, none of the three female specimens collected in primary forest tested positive for *Leishmania* spp. DNA. The taxonomic revision by Sábio et al. clarified that several taxa previously grouped under *Pa. shannoni* actually represent distinct species (*Pa. limai, Pa. bigeniculata,* *Pa. ribeirensis* and *Pa. baratai* sp. n.) each with specific geographic ranges in Brazil and other Neotropical regions [121,122]. Consequently, historical records attributed to *Pa. shannoni* in southern and central Brazil, such as those reported by Dorval et al., may include misidentified members of this complex [123]. Extending the confirmed distribution of *Pa. shannoni* sensu stricto beyond its core range (from the southern United States to northern South America) will require the integration of morphological, molecular, and ecological data to minimize taxonomic uncertainty. Although *Pa. shannoni* has occasionally been found infected with *Leishmania* (*Viannia*) spp. in various localities, its role as a vector remains secondary or incidental compared to species such as *Ny. whitmani* or *Mg. migonei.* The recognition of cryptic diversity within the *Shannoni* complex further emphasizes the need for updated assessments of both distribution and vector competence, particularly in areas where sympatric *Viannia* species circulate. In such regions, genetic exchange—such as that demonstrated by Stocco Lima et al. for *L. guyanensis*/ *L. shawi* hybrids—may additionally complicate eco-epidemiological interpretations [124].

*Ev. thermitophila* is distributed across Brazil, Bolivia, and Argentina [125] and has been recently recorded in Paraguay, with a male specimen captured in the San Pedro department [6]. This species is typically associated with the burrows of wild animals and is found in rock crevices or in proximity to farm animals in rural areas. Our study represents the first finding of *Ev. thermitophila* in Canindeyú, where it was collected in the primary forest, extending its known geographic range but without evidence of positivity to *Leishmania* spp. DNA. However, previous research in Brazil has documented the presence of *L. infantum* DNA [78] and *L. guyanensis* DNA [126], reflecting the potential of this species to serve as a host for these parasites.

The RNBM harbors the largest and most protected remnant of the Atlantic Forest, composed of late-stage secondary forest with minimal human impact over the past 35 years [57]. The activities allowed in the RNBM include traditional hunting by the Ache indigenous people, limited biological research, and ecotourism. During our study, we collected sandflies in five vegetation classes present in the RNBM: high forest, bamboo and liana forest, bamboo understory, and riparian vegetation [40]. However, the traps were placed in two well-differentiated forest zones: the primary forest, which includes high forest, bamboo, riparian vegetation, and bamboo understory, and the secondary forest, composed of high forest, bamboo, and lianas. The bamboo forest is characterized by having few trees and a predominance of bamboo, which can reach heights of 10–15 meters. In contrast, the liana forest features abundant lianas that dominate the understory, while in the high forest, tree heights reach around 20 meters, with an open understory [57].

The results of this study confirm the association of a higher abundance of sandflies in areas with greater vegetation cover and their decrease as soil cover or agricultural areas increase. Therefore, the largest number of specimens was collected in the secondary forest, specifically in areas of liana and bamboo forests, followed by the primary forest where the vegetation consists of tall forests and bamboo. The main difference between primary and secondary forests lies in the greater human presence in the latter, including ecotourism areas. These ecotourism practices can facilitate the interaction between domestic animals, humans, and wildlife, increasing the risk of disease transmission [127].

The collection area in degraded soil was conducted on a farm near the entrance of the RNBM, characterized by meadows, grasslands, and degraded areas. In this area, we obtained a low quantity and diversity of sandflies, with the highest number captured in a trap located on a forest island within a pasture frequented by cows, near the primary forest of the RNBM. This behavior is not surprising, considering that sandflies are originally wild. The distribution and abundance of species are directly influenced by the presence of forested areas, as observed in a study on the faunal diversity of sandflies in southern Brazil, which established a correlation between insect richness and the existence of forested areas [59].

Although the anthropized species *Ev. evandroi* and *Ny. neivai* showed low capture rates, they showed DNA rates of 4.3% for *L. infantum* and *L. amazonensis,* respectively, suggesting a significant risk of transmission for nearby peri-domestic and domestic populations. On the other hand, the wild species *Pi. monticola* and *Br. brumpti*, attracted to light and typical of edge areas, also tested positive for *L. infantum* and *L. amazonensis* DNA, with DNA rates of 18.8% and 17.4%, respectively. These species could play a role in the transmission of leishmaniasis, especially in areas where human intervention has altered the forest, potentially facilitating their movement towards more populated areas.

However, certain methodological factors may have influenced the detection of the main vectors of *L. amazonensis* and *L. infantum*. The sampling strategy relied primarily on CDC light traps placed in sylvatic environments, with limited peridomestic coverage. This approach may have underestimated *Lutzomyia* species, which are often concentrated in peridomiciliary and animal shelter areas, particularly in anthropized environments [128]. Similarly, *Bichromomyia flaviscutellata*–the established vector of *L. amazonensis* in Brazil, though not yet reported in Paraguay–shows low attraction to light traps and exhibits cryptic behavior, typically inhabiting rodent burrows and humid forest soil microhabitats [129]. Its apparent absence in our captures may therefore reflect sampling bias rather than true ecological scarcity. Future studies should adopt a broader trapping strategy, including emergence traps, aspiration near rodent nests, and more intensive peridomestic sampling, to improve vector detection and provide a more accurate representation of transmission foci diversity.

Although several of the dogs evaluated showed evident clinical signs of leishmaniasis, all tested negative when analyzed using the immunochromatographic rk39 test. This result, though initially puzzling, aligns with previously documented limitations of this test in studies conducted in Brazil [130,131] and Argentina [132]. These studies have shown that the low sensitivity of the rk39 test makes it difficult to accurately detect *L. infantum* infections in canines, particularly those with mild clinical manifestations or low parasitic loads. This limitation could be even more significant if the presence of other *Leishmania* species that can infect canines [20] is considered, as the test is specifically designed to detect species of the *L. donovani* complex. Therefore, exclusive dependence on this diagnostic tool in endemic areas could lead to an underestimation of the real prevalence of canine leishmaniasis, highlighting the need to use complementary diagnostic approaches for a more thorough evaluation. In this study, molecular assays were not performed because we only obtained authorization from the dog owners for the extraction of peripheral blood, which does not represent the most suitable tissue for detecting *Leishmania* spp. However, the ectoparasites collected from these dogs revealed a notably high positivity for *L. amazonensis* DNA when analyzed using molecular techniques. This finding is particularly significant, as although sandflies are recognized as the exclusive biological vectors of *Leishmania* spp., speculation has begun regarding potential secondary modes of transmission, particularly in areas where cases of leishmaniasis have been reported without confirmed presence of known vectors [26,27,111,133], or even in the absence of sandflies [28]. In this context, ticks and fleas, highly specialized hematophages, have been identified as potential transmitters [36,130,134], as they feed on a variety of mammals, including humans, livestock, dogs, cats, rabbits, squirrels, rats, and mice [135]. The transmission of *Leishmania* spp. by infected fleas or ticks, accidentally ingested by dogs or other mammals, has also been suggested in previous studies [35,136], as occurs with the protozoan parasite *Hepatozoon canis*, which is effectively transmitted through the ingestion of infected ticks [137].

Analysis of environmental variables revealed that the abundance and species composition of phlebotomine sandflies at the sentinel sites were strongly influenced by both land cover characteristics and local climatic conditions, displaying spatial patterns consistent with those reported for other Neotropical regions. The negative association between sandfly abundance and agricultural land cover within 25–100 m suggests that areas subject to greater anthropogenic disturbance provide less suitable conditions for sustaining sandfly populations. This pattern is consistent with the findings of Rêgo et al. in the Xakriabá Indigenous Reserve [138], where vegetation loss and soil compaction reduced local diversity, and with those of Pereira et al. who reported lower species richness and abundance in urbanized areas of Itaúna, Minas Gerais [139].

Conversely, the positive correlations observed between sand fly abundance and forest cover at 50 and 100 m underscore the importance of vegetation in maintaining suitable microhabitats by moderating temperature and increasing humidity. Silva et al. demonstrated that both primary and secondary forest fragments harbor significantly greater sand fly richness and density compared with built-up zones, emphasizing their role as ecological refuges in urban and periurban contexts [140]. In our study, secondary forest showed the highest total abundance, reinforcing the hypothesis that regenerating environments provide an optimal balance of organic matter, shading, and blood sources conditions particularly favorable for species such as the *Ev. cortelezzii* complex and *Ny. neivai*.

Discriminant analysis further revealed marked differences among habitat types, with some species overlap between primary and secondary forests. This pattern reflects the ecological plasticity of certain taxa. Fernandes et al. reported similar results in Mato Grosso do Sul, where generalist species such as *Lu. longipalpis* and *Mg. migonei* successfully colonized both natural and periurban environments [141]. Such coexistence may be linked to the simultaneous availability of shelter, organic matter, and hosts, as well as tolerance to variable microclimatic conditions.

Regarding climatic variables, the positive correlation between mean temperature and sand fly abundance supports previous studies associating higher temperatures with increased development rates, survival, and dispersal capacity [141–143]. Oliveira et al. documented a direct relationship between temperature and *Lu. longipalpis* density, indicating that warmer periods promote reproductive and feeding activity [142]. Similarly, Carvalho et al. demonstrated that rising temperatures expand the ecological niche of *Bi. flaviscutellata*, enabling its projected southward range expansion in South America under climate change scenarios [143].

The weak but negative correlation with minimum humidity suggests that environmental desiccation may limit sand fly abundance, particularly during immature stages that depend on moist, organic-rich substrates. This finding agrees with Silva et al., who reported abundance declines during drier months [140], and Fernandes et al., who identified relative humidity as a key determinant of population success [141]. The absence of a significant relationship with wind speed is consistent with Oliveira et al., who observed a limited effect of wind in vegetated areas where canopy cover reduces its impact on sand fly flight activity [142].

The species–habitat associations observed here—*Ps. shannoni* and *Ps. lanei* in primary forest, *Mg. migonei* and *Ev. evandroi* in farm areas—mirror the ecological partitioning described by Rêgo et al. [138] and Pereira et al. [139]. In these studies, specialist forest species were distinguished from generalist taxa adapted to modified landscapes. This differentiation suggests a successional gradient in community structure, in which secondary forests act as transitional zones facilitating coexistence and dispersal of medically important species.

Taken together, our findings, supported by recent literature, reinforce the role of environmental and climatic factors in shaping sand fly population dynamics in tropical settings. Forest fragments—particularly secondary ones—represent key habitats for the persistence and expansion of vector species and may play a central role in *Leishmania* transmission across fragmented landscapes. Therefore, entomological surveillance in transitional and periurban zones, combined with fine-scale monitoring of vegetation cover and local climate, should be prioritized as part of integrated leishmaniasis control strategies in South America.

## 5 Conclusions

The results presented in this study reveal a remarkable diversity of sandfly species that could be involved in the transmission of *Leishmania* spp. in this remnant of the Atlantic Interior Forest, some of which are reported for the first time in the region. The detection of *L. amazonensis* and *L. infantum* in the absence of their traditional vectors raises questions about new transmission dynamics, especially considering that several of the species captured and positive for *Leishmania* spp. DNA have previously been identified as potential vectors of LTA and LV. The high number of positive specimens captured in the secondary forest highlights its relevance in terms of risk, as it could facilitate the transmission of *Leishmania* spp. to tourists and people involved in educational and scientific activities in the area. Furthermore, the high detection of *L. amazonensis* DNA in ectoparasites collected from dogs in the indigenous community represents a relevant finding, as these arthropods could facilitate parasite transmission, with key implications regarding the role of dogs as reservoirs of the pathogen. It is crucial to incorporate molecular methods in future studies to detect and identify the pathogen in both domestic and wild reservoirs, which will facilitate a more detailed understanding of the complex transmission dynamics of the disease. In this sense, addressing the transmission of leishmaniasis in wild environments requires adopting strategies beyond traditional approaches, establishing entomo-epidemiological surveillance as a strategic tool within the One Health paradigm.

## Supporting information

S1 Table Sandfly species recorded per trap in different ecotopes of the RBBM, Canindeyú Department, Paraguay, between October 2020 and October 2021. (DOCX)

S2 Table Correlation analysis between environmental variables and the abundance of sandflies captured in the RBBM, Canindeyú, Paraguay. (DOCX)
