# Supplementary material for: The diversity of sandflies (Psychodidae: Phlebotominae) and the presence of Leishmania spp. DNA in potential vectors of the Mbaracayú Forest Biosphere Reserve, Canindeyú, Paraguay: New records and findings
Source: PLoS Negl Trop Dis. 2025 Dec 10;19(12):e0013806. doi: 10.1371/journal.pntd.0013806 (PMC12694836; doi:10.1371/journal.pntd.0013806)
Supplement: S1 Table — (DOCX) [file pntd.0013806.s001.docx]

**S1 Table:** Sand fly species recorded per trap in different ecotopes of the RBBM, Canindeyú Department, Paraguay, between October 2020 and October 2021.

| **Light trap codes per zone** | **Geographical coordinates (UTM)** | | **Sandfly species** |
| --- | --- | --- | --- |
|  | **E (m)** | **N (m)** |  |
| A1 | 648982 | 7330279 | *Ev. cortelezzi* complex |
| A2 | 648930 | 7330401 | *Ev. cortelezzii* complex  *Brumptomyia brumpti*  *Pintomyia monticola* |
| A3 | 649035 | 7330410 | *Ev. cortelezzii* complex  *Brumptomyia brumpti*  *Pintomyia montícola*  *Evandromya evandroi* |
| A4 | 649282 | 7330294 | *Ev. cortelezzii* complex  *Brumptomyia brumpti*  *Pintomyia monticola*  *Psathyromyia lanei*  *Micropygomyia quinquefer*  *Nyssomya neivai* |
| A5 | 649160 | 7330296 | *Ev. cortelezzii* complex  *Brumptomyia brumpti*  *Pintomyia montícola*  *Psathyromyia lanei*  *Evandromya evandroi*  *Brumptomyia avellari*  *Micropygomyia quinquefer*  *Nyssomya neivai* |
| A6 | 649452 | 7330376 | *Ev. cortelezzii* complex  *Brumptomyia brumpti*  *Pintomyia montícola*  *Psathyromyia lanei*  *Evandromya evandroi*  *Brumptomyia avellari*  *Nyssomya neivai* |
| A7 | 649435 | 7330507 | *Ev. cortelezzii* complex  *Brumptomyia brumpti*  *Pintomyia montícola*  *Psathyromyia lanei*  *Evandromya evandroi*  *Nyssomya neivai*  *Migonemyia migonei* |
| A8 | 649374 | 7330231 | *Ev. cortelezzii* complex *Brumptomyia brumpti*  *Pintomyia montícola*  *Evandromya evandroi*  *Micropygomyia quinquefer*  *Migonemyia migonei* |
| A9 | 649359 | 7330379 | *Ev. cortelezzii* complex  *Brumptomyia brumpti*  *Pintomyia montícola*  *Evandromya evandroi*  *Brumptomyia avellari*  *Brumptomyia guimaraesi* |
| A10 | 648901 | 7330311 | *Ev. cortelezzii* complex  *Brumptomyia brumpti*  *Pintomyia montícola*  *Psathyromyia lanei*  *Evandromya evandroi* |
| B1 | 648062 | 7330957 | No captured |
| B2 | 648060 | 7331129 | *Ev. cortelezzii* complex  *Brumptomyia brumpti* |
| B3 | 647990 | 7331251 | *Ev. cortelezzii* complex  *Brumptomyia brumpti*  *Pintomyia montícola*  *Migonemyia migonei* |
| B4 | 648065 | 7330533 | *Ev. cortelezzii* complex  *Brumptomyia brumpti*  *Pintomyia monticola*  *Psathyromyia lanei* |
| B5 | 648133 | 7330462 | *Ev. cortelezzii* complex  *Pintomyia montícola*  *Psathyromyia lanei* |
| B6 | 648095 | 7330745 | No captured |
| B7 | 648460 | 7330335 | *Ev. cortelezzii* complex  *Pintomyia montícola*  *Evandromya evandroi*  *Brumptomyia avellari*  *Brumptomyia guimaraesi*  *Pintomyia montícola* |
| B8 | 648267 | 7330403 | *Ev. cortelezzii* complex  *Pintomyia montícola*  *Evandromya evandroi*  *Brumptomyia avellari*  *Brumptomyia guimaraesi*  *Pintomyia montícola*  *Micropygomyia quinquefer* |
| B9 | 648183 | 7330883 | *Ev. cortelezzii* complex  *Brumptomyia brumpti*  *Evandromya evandroi* |
| B10 | 647968 | 7331406 | *Ev. cortelezzii* complex  *Pintomyia montícola*  *Evandromya evandroi*  *Brumptomyia avellari*  *Pintomyia montícola*  *Micropygomyia quinquefer*  *Evandromyia termitophila*  *Psathyromyia shannoni* |
| C1 | 647172 | 7333608 | *Ev. cortelezzii* complex |
| C2 | 647197 | 7333641 | *Brumptomyia brumpti* |
| C3 | 647180 | 7333593 | *Ev. cortelezzii* complex |
| C4 | 647193 | 7333566 | *Ev. cortelezzii* complex  *Brumptomyia brumpti* |
| C5 | 647160 | 7333545 | *Brumptomyia brumpti* |
| C6 | 647123 | 7333452 | *Brumptomyia brumpti* |
| C7 | 647228 | 7333539 | No captured |
| C8 | 647081 | 7333633 | No captured |
| C9 | 646911 | 7333621 | No captured |
| C10 | 646628 | 7333567 | *Ev. cortelezzii* complex  *Brumptomyia brumpti*  *Pintomyia monticola* |
