# Supplementary material for: The diversity of sandflies (Psychodidae: Phlebotominae) and the presence of Leishmania spp. DNA in potential vectors of the Mbaracayú Forest Biosphere Reserve, Canindeyú, Paraguay: New records and findings
Source: PLoS Negl Trop Dis. 2025 Dec 10;19(12):e0013806. doi: 10.1371/journal.pntd.0013806 (PMC12694836; doi:10.1371/journal.pntd.0013806)
Supplement: S2 Table — (DOCX) [file pntd.0013806.s002.docx]

**S2 Table.** Correlation between environmental variables and the total number of sandflies captured in the different ecotopes of the RBBM, Canindeyú, Paraguay.

| **Correlation between environmental variables and the total number of sandflies** | | | | |
| --- | --- | --- | --- | --- |
| **Environmental variables** | | **Statistical significance** | | |
|  |  | ***r*** | ***r2*** | ***P*** |
| Temperature | | 0.2350 | 0.0552 | 0.0004 |
| Temperature (min) | | 0.3851 | 0.3325 | 0.2480 |
| Temperature (max) | | 0.2130 | 0.3287 | 0.5180 |
| Relative humidity | | -0.0995 | 0.0099 | 0.1427 |
| Humidity (min) | | 0.1796 | 0.0831 | 0.0032 |
| Humidity (max) | | 0.2719 | 0.0991 | 0.7840 |
| Wind speed | | -0.0949 | 0.0090 | 0.1622 |
| **Buffer distance (meters)** | **Vegetation indices** | ***r*** | ***r2*** | ***P*** |
| 25 | NDVI 1 | 0.2928 | 0.0857 | 1,1027E-05 |
|  | NDVI 2 | 0.2481 | 0.0615 | 0.0002 |
|  | NDWI 1 | -0.2153 | 0.0463 | 0.0013 |
|  | NDWI 2 | -0.2042 | 0.0417 | 0.0024 |
| 50 | NDVI 1 | 0.2930 | 0.0858 | 1.0895E-05 |
|  | NDVI 2 | 0.2375 | 0.0564 | 0.00040 |
|  | NDWI 1 | -0.1806 | 0.0326 | 0.0074 |
|  | NDWI 2 | -0.2051 | 0.0420 | 0.00233 |
| 100 | NDVI 1 | 0.2922 | 0.0854 | 1.1512E-05 |
|  | NDVI 2 | 0.2450 | 0.0600 | 0.0003 |
|  | NDWI 1 | -0.2302 | 0.0530 | 0.0006 |
|  | NDWI 2 | -0.2020 | 0.0408 | 0.0027 |
| **Land cover percentage (meters)** | **Land covers** | ***r*** | ***r2*** | ***P*** |
| 25 | Bare soil | -0.1433 | 0.0205 | 0.1261 |
|  | Forest | 0.1039 | 0.0107 | 0.1261 |
|  | Water | 0 | 0 | 0 |
|  | Agricultural land | -0.1533 | 0.0235 | 0.0235 |
| 50 | Bare soil | -0.2239 | 0.0501 | 0.0009 |
|  | Forest | 0.2558 | 0.0654 | 0.0001 |
|  | Water | 0 | 0 | 0 |
|  | Agricultural land | -0.1863 | 0.0347 | 0.0058 |
| 100 | Bare soil | -0.2261 | 0.0511 | 0.0007 |
|  | Forest | 0.2591 | 0.0671 | 0.0001 |
|  | Water | -0.0573 | 0.0033 | 0.3995 |
|  | Agricultural land | -0.1897 | 0.0360 | 0.0049 |
